# Malignant Eccrine Spiradenoma of the Posterior Scalp: An Odd Presentation

**DOI:** 10.7759/cureus.40033

**Published:** 2023-06-06

**Authors:** Samuel Lichtman-Mikol, Aria Kieft, Rafic Beydoun, Michael Joiner, Steven Miller

**Affiliations:** 1 Department of Oncology, Wayne State University School of Medicine, Detroit, USA; 2 Department of Radiation Oncology, Wayne State University Detroit Medical Center, Detroit, USA; 3 Department of Pathology, Wayne State University Detroit Medical Center, Detroit, USA

**Keywords:** locally aggressive, malignant proliferation, surgery, postoperative radiation therapy, rare skin disease

## Abstract

Malignant eccrine spiradenoma is a rare cutaneous adnexal neoplasm and is often a result of the malignant transformation of a benign eccrine spiradenoma. A woman without a history of skin cancer presented with a mass on her posterior scalp. An excisional biopsy was obtained, and histology was consistent with eccrine spiradenocarcinoma with the lesion extending to all margins of the excision specimen. Physical exam and imaging did not reveal lymph node involvement or distant spread of disease. It was recommended that the patient undergo wide local excision.

## Introduction

Malignant eccrine spiradenoma (MES) is a rare cutaneous adnexal neoplasm and is often a result of the malignant transformation of a benign eccrine spiradenoma (ES). A woman without a history of skin cancer presented with a mass on her posterior scalp. An excisional biopsy was obtained, and histology was consistent with eccrine spiradenocarcinoma with the lesion extending to all margins of the excision specimen. Physical exam and imaging did not reveal lymph node involvement or distant spread of disease. It was recommended that the patient undergo wide local excision.

MES, or spiradenocarcinoma, is an extremely rare sweat gland tumor typically resulting from a malignant transformation from a benign ES [1-5], though, less commonly, has also been described to develop de novo [6,7]. MES was first described by Dabska in 1972 [8], and just over 100 cases have been reported between then and now [9,10]. The overall incidence of head and neck sweat gland adenocarcinoma (HNSGA) is 0.036 per 100,000 people [11], and MES accounts for roughly 0.005% of all skin tumors [12]. While MES has developed in patients from ages ranging between 21 and 92 years, the peak incidence is 59 years of age, with both sexes similarly affected [13,14]. MES is aggressive and has a poor prognosis after metastasis, with median overall survival of around one year [9,15]. MES has been shown to metastasize to bone, lung, liver, or brain most often [9,10]. We present the case and management of a woman in her mid-70s with MES localized to her posterior scalp.

## Case presentation

**Figure 1:**
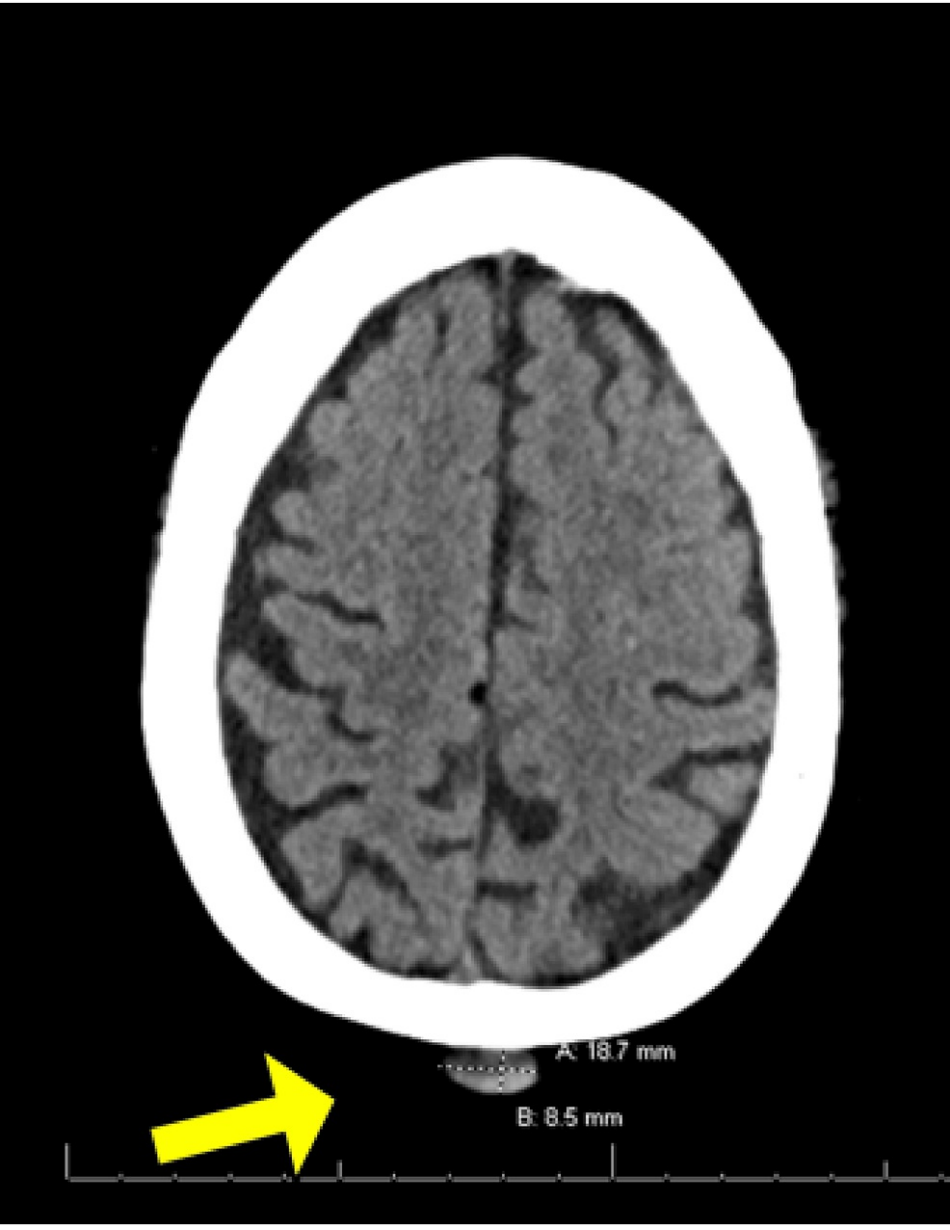
Image of a posterior scalp lesion

**Figure 2:**
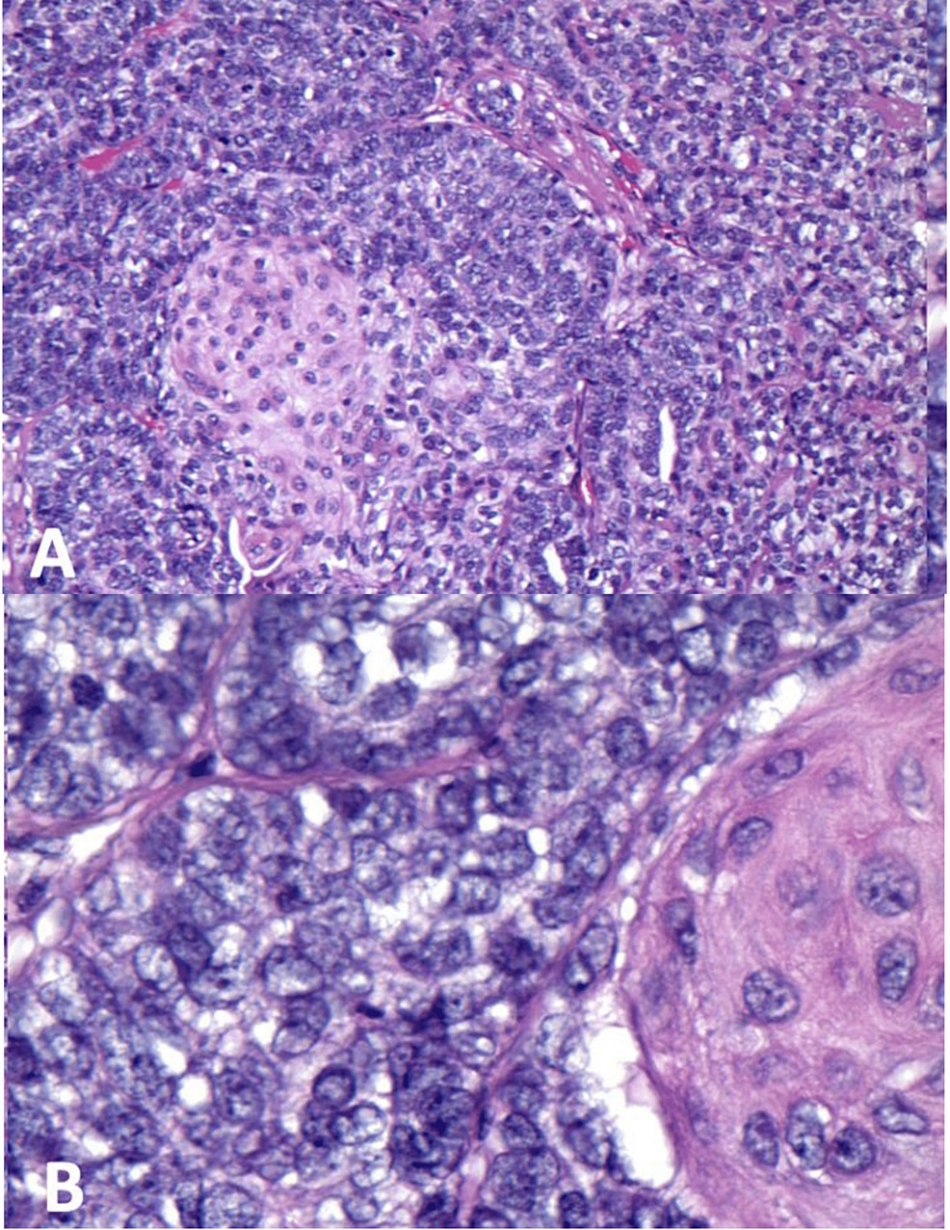
(A) Section reveals loss of the dual cell population, with the proliferation of small cells with hyperchromatic/vesicular nuclei, high N/C ratio, minimal cytoplasm, moderate to severe cytological atypia, increased mitotic activity, and focal squamoid differentiation. (B) High power of the previous image with squamoid differentiation on the right side and mitosis in the center.

An elderly woman with a medical history of obesity, diabetes mellitus, chronic kidney disease, and rheumatoid arthritis first noticed a mass on her vertex scalp in 2019, as visualized by computed tomography (CT) in Figure 1. The mass persisted and she sought out a dermatologist in November 2021. She then underwent an excisional biopsy and a histopathologic review demonstrated eccrine spiradenocarcinoma, as seen in Figure 2. The lesion was noted to extend to all margins of the biopsy specimen. CT scans of her head and neck, chest, abdomen, and pelvis were performed without contrast due to the patient’s poor renal function, and no evidence of metastasis was found. Given her medical history, the patient and her daughter were concerned about the patient’s ability to tolerate surgical resection and removal of lymph nodes. Radiation therapy was discussed as a possible curative option or as part of a palliative plan. The patient agreed to have her case, including radiologic and pathologic studies, be presented at the multidisciplinary tumor board. The team consisted of medical oncology, radiation oncology, surgery, speech pathology, and dental and oral maxillofacial prosthodontics. The final recommendation of the tumor board was wide local excision of positive margins.

The patient first presented to her dermatologist, who initially thought the lesion was an ES. They then performed a wide local excision and sent three specimens to pathology for review. Dermatopathology reported nodular proliferation of cells, with islands of cells with darker cells lining the periphery and lighter cells on the inside of the lobules. There were scattered mitotic figures and areas of keratinization and the lesion had a large infiltrating architecture. The findings were consistent with eccrine spiradenocarcinoma with the lesion extending to all margins of the specimen, indicating positive margins and concern for metastasis. Six months after excision of the lesion, CT scans of the patient’s head and neck, chest, abdomen, and pelvis were taken and did not demonstrate evidence of metastasis.

The initial differential diagnosis consisted of lipoma, pilar cyst, basal cell carcinoma (BCC), squamous cell carcinoma (SCC), Merkel cell carcinoma (MCC), angiosarcoma, and metastatic carcinoma. All the above diagnoses were ruled out once an excisional biopsy was performed to confirm histopathology.

Seven months after the patient initially underwent an excisional biopsy, she contracted COVID-19 and her overall health declined. Eight months after the initial excisional biopsy, the patient was contacted by phone and was still in poor health due to post-COVID symptoms complicated by her chronic medical problems. The patient’s daughter, and caretaker, had not observed any recurrence of skin lesions or swollen lymph nodes and described the initial incision as well-appearing. The physicians of the tumor board still recommended additional wide local excision, but the patient had not seen her dermatologist for treatment due to poor mobility. The patient’s daughter was again contacted 11 months after the initial biopsy and the team was notified of the patient’s death.

## Discussion

ES was first described by Kersting and Helwig in 1956 [16], as a benign tumor commonly hard and 1-2cm in size often located on the trunk or extremities, and can be associated with pain and tenderness [17]. Then, in 1972, Dabska published the first case regarding MES, a malignant transformation of ES [8] which accounts for only 0.005% of all skin tumors [12]. The malignant transformation is a slow progression from a long-standing benign lesion, often occurring after 20-30 years [15,18]. The pathological examination may reveal a transition zone where a region of undifferentiated malignant cells containing numerous mitotic figures arises in a field of more benign-appearing cells [15]. The presence of benign ES tissue adjacent to, or surrounding, the malignant proliferation of MES is characteristic of the diagnosis of MES [10].

Other etiologies that should be considered in the differential diagnosis for this patient included a lipoma which is commonly occurring and can present as a soft, non-tender nodule. This was ruled out since the lesion in our patient was firm and tender to palpation. The pilar cyst was also considered as it is commonly occurring and presents as a firm and slow-growing nodule, though usually, it is not tender to palpation unless it ruptures.

BCC and SCC must be considered as they are the most common forms of skin cancer and can present with non-healing ulceration and progressive growth. MCC and angiosarcoma are rapidly growing and can present as a skin-colored or blue-red nodule with or without ulceration. Metastatic carcinoma often presents as a firm nodule that varies in color (skin-colored, red, violaceous, hyperpigmented). This can be identified with excisional biopsy.

Physical exam often reveals erythema, ulceration, tenderness, bleeding, or growth on serial exams and/or the development of satellite lesions from a longstanding ES [3,9,13,19,20]. Due to the histopathological characteristics of MES, where malignant cells are often surrounded by benign ES tissue, skin surface changes may not be evident for some time. However, when the patient does present with recent skin changes such as ulceration overlying a long-standing ES nodule, it is likely that the malignant cells have already infiltrated through the surrounding benign ES tissue. Extracapsular extension beyond the ES capsule is indicative of more aggressive behavior and is associated with a higher risk of metastasis.

Although there are no published guidelines for intervention, the current standard of care is to perform wide local excision with 1-cm margins with a depth down to the fascia, or Mohs micrographic surgery [6,10,21]. It has been found that wide local surgical excision is the definitive treatment for patients with localized disease and a tumor-free margin, as one meta-analysis found 35 out of 35 patients remained disease free with a mean follow-up period of 33 months [9]. They also found that six out of seven patients who had positive lymph node spread and were treated with surgical excision plus lymph node dissection remained disease free at a mean follow-up of 47 months [9]. Given poor outcomes when diagnosed after metastatic spread, the literature indicates early detection and localized therapy is imperative [9]. The literature review has suggested that only evidence of distant metastasis is associated with fatal outcomes [9,10,20], where the most common sites of metastasis beyond regional lymph nodes are lungs, brain, bone, and liver [7]. This depicts its aggressive and dangerous behavior, especially considering the mean time to death with unsuccessfully treated cases ranging from less than one year of survival to 16 months [9,15].

As surgical interventions are most commonly used to treat localized MES, the role of adjuvant radiotherapy or chemotherapy is not well understood, especially since MES has been described to be radioresistant [11]. Despite not being typically utilized as initial therapy, radiotherapy has been used for palliative care [22], as well as to treat positive margins and gross residual tumors [23-27]. In one reported case, the patient diagnosed with MES of the thigh with regional lymph node metastasis was treated with radical surgical excision with lymph node dissection followed by a total dose of 50 Gy to both the tumor bed and inguinal lymph nodes using external beam radiotherapy (EBRT), though fractionation was not reported [26]. The patient then underwent six cycles of chemotherapy using carboplatin and paclitaxel, allowing the patient to remain disease-free three years post-treatment. Another case of MES occurred when a longstanding lesion on the leg began to ulcerate and grow, which was treated by surgical excision and lymph node dissection, followed by radiotherapy of a total dose of 59.4 Gy in 1.8 Gy fractions [27]. The inguinal and pelvic lymph nodes were also treated with a total dose of 45 Gy in 1.8 Gy fractions. The patient remained in remission until nine months later when the tumor recurred at the original location. Given the heterogeneity of the location of the disease, disease progression, and cell surface receptor presence within this disease, chemotherapies have also been used as adjuvant treatment with limited success [15,27-29].

Due to the rarity of this disease and the limited number of cases, meta-analyses are the most comprehensive literature available to review the best treatment options, since to our knowledge, no treatment guidelines currently exist [9]. Our patient presented with positive margins status post excisional biopsy and was recommended additional excision with wide margins. Due to our patient’s poor health status recovering from COVID-19 in addition to her other comorbidities, she planned to seek a second local excision once she could ambulate more easily. However, the patient, unfortunately, passed away prior to this. Given the aggressiveness of this rare disease, early intervention and close follow-up are necessary to prevent and limit tumor recurrence and/or metastasis.

## Conclusions

In conclusion, MES is a rare skin disease and can arise de novo or from the malignant transformation of benign ES. Local MES can be successfully treated with wide local excision, while MES with lymph node extension can be successfully treated with wide local excision plus lymph node dissection. The role of radiation therapy in this situation is not clearly defined.

MES is aggressive and a disease that has distant metastases has been associated with poor outcomes, thus early diagnosis, treatment, and close follow-up are imperative. Treatment for this disease is not clearly defined but surgery should be the primary treatment. Adjuvant radiation therapy can be considered for patients with positive margins and or node-positive disease.
